# Supplementary material for: Nutritional and Morphofunctional Assessment in a Cohort of Adults Living with Cystic Fibrosis with or Without Pancreatic Exocrine and/or Endocrine Involvement
Source: Nutrients. 2025 Jun 20;17(13):2057. doi: 10.3390/nu17132057 (PMC12251311; doi:10.3390/nu17132057)
Supplement: Supplementary file 1 [file nutrients-17-02057-s001.zip › nutrients-3687542-supplementary.pdf]

Supplementary Table S1. Demographic and main characteristics related to their disease in a cohort of adult CF patients, stratified by malnutrition diagnosis using GLIM criteria.

Variable	Malnourished (n = 48)	Not malnourished (n = 53)	p Between Malnutrition
Sex (women)	23 (47.9%)	21 (39.6%)	0.401
Age (years)	33 (24–40)	31 (26–41)	0.932
>45 years old	2 (4.2%)	9 (17%)	0.039
Time of evolution (years)	28 (20-37)	23 (13–18)	0.228
Onset of symptomatology in infancy	38 (79.2%)	37 (69.8%)	0.261
Mutation			
Minimal function	29 (60.4%)	21 (39.6%)	0.037
Minimum/residual function	19 (39.6%)	29 (54.7%)	0.128
Residual function	0 (0%)	3 (5.7%)	0.094
ΔF508 mutation	37 (77.1%)	41 (77.4%)	0.974
Homozygous	16 (33.3%)	13 (24.5%)	0.329
Heterozygous	21 (43.8%)	28 (52.8%)	0.362
FEV1	68 (44-82)	79 (63-92)	0.178
FVC	79 (68-93)	93 (82-102)	0.052
FEV1/FVC	66 (54-78)	71 (62-77)	0.348
Exocrine pancreatic insufficiency	36 (75%)	28 (52.8%)	0.021
Endocrine pancreatic insufficiency	25 (52.1%)	19 (35.2%)	0.1
CF-related diabetes (CFRD)	15 (31.3%)	13 (24.5%)	0.451
CF-related prediabetes	9 (18.8%)	5 (9.4%)	0.176
CF-related indeterminate glycemia	1 (2.1%)	1 (1.9%)	0.944

n: absolute frequency; CF: cystic fibrosis; CFRD: CF-related diabetes; FEV_1: Forced expiratory volume in 1 second; FVC: Forced vital capacity; GLIM: Global Leadership Initiative in Malnutrition.
